# Assessing Significance in High-Throughput Experiments by Sequential Goodness of Fit and *q*-Value Estimation

**DOI:** 10.1371/journal.pone.0024700

**Published:** 2011-09-09

**Authors:** Antonio Carvajal-Rodriguez, Jacobo de Uña-Alvarez

**Affiliations:** 1 Departamento de Bioquímica, Genética e Inmunología, Facultad de Biología, Universidad de Vigo, Vigo, Spain; 2 Departamento de Estadística e Investigación Operativa, Facultad de Ciencias Económicas y Empresariales, Universidad de Vigo, Vigo, Spain; Rutgers University, United States of America

## Abstract

We developed a new multiple hypothesis testing adjustment called SGoF+ implemented as a sequential goodness of fit metatest which is a modification of a previous algorithm, SGoF, taking advantage of the information of the distribution of *p*-values in order to fix the rejection region. The new method uses a discriminant rule based on the maximum distance between the uniform distribution of *p*-values and the observed one, to set the null for a binomial test. This new approach shows a better power/pFDR ratio than SGoF. In fact SGoF+ automatically sets the threshold leading to the maximum power and the minimum false non-discovery rate inside the SGoF' family of algorithms. Additionally, we suggest combining the information provided by SGoF+ with the estimate of the FDR that has been committed when rejecting a given set of nulls. We study different positive false discovery rate, pFDR, estimation methods to combine *q*-value estimates jointly with the information provided by the SGoF+ method. Simulations suggest that the combination of SGoF+ metatest with the *q*-value information is an interesting strategy to deal with multiple testing issues. These techniques are provided in the latest version of the SGoF+ software freely available at http://webs.uvigo.es/acraaj/SGoF.htm.

## Introduction

Multiple hypothesis testing has become an important issue since the advent of “omic” technologies: genomics, proteomics, transcriptomics etc. Usually it involves the simultaneous testing of thousands of hypotheses producing a set of significant *p*-values. The later may be indicating some kind of true effect for each test. By true effect we mean, depending on the kind of experiments, an increased expression of a gene, or quantity of RNA, protein and so on. There are several methods controlling the family wise error rate (FWER) with the aim to minimize the type I error, i.e. the problem of detecting effects which are not true ones. Unfortunately, minimizing type I error increases type II error, that is, diminishes the statistical power to detect true effects. An interesting alternative is to control the false discovery rate, FDR [1], which is the expected proportion of false discoveries among the total ones i.e. the expected proportion of the rejected null hypotheses which are erroneously rejected. When computing the FDR, two strategies can be followed [2], [3]. First, fixing an FDR level of acceptance, say 5%, and then detecting the rejection region of interest i.e. the widest region with associated FDR below the fixed level. The second strategy has been proposed by Storey and Tibshirani [4] and aims to fix the rejection region and then to estimate the FDR over that region. This strategy also provides the estimation of the *q*-values linked to each test, i.e. the expected proportion of false positives incurred if we call a given test significant [2]. However, the use of the *q*-values does not provide an automatic procedure to detect true effects while informing about the probability of committing a false discovery. The computation of *q*-values implies a previous estimation of the fraction of tests with true null distribution [4]. It is worth mentioning that the so called pFDR, i.e. FDR conditional upon having rejected one or more hypotheses, can not be controlled when the probability of having an effect is low [2], [5] or even when the *p*-values are obtained from low-sample size tests [6]. The above problems are still more serious when considering that, under realistic sample sizes, the FDR controlling methods increase type II error if the number of tests is high and the size of the effects are weak i.e. the higher the number of tests the lower the power to detect true effects [6].

The obvious goal when performing multiple testing adjustments is to detect as many true positives as possible while maintaining the false ones below a desired threshold. Therefore, for a fixed percentage of existing effects, the higher the number of tests performed, the higher the number of true positives that should be detected. In a previous work [6] we have proposed an exact binomial meta-test as a new multiple testing adjustment which holds the desirable property of increasing power with the number of tests. The binomial meta-test compares the observed and expected proportions of *p*-values falling below threshold γ (often γ = 0.05) and makes a decision about the number of effects accordingly. The method was shown to behave especially well with weak-moderate alternative hypothesis, high number of tests and low sample size. Since the SGoF method only controls for FWER in the weak sense i.e. under the complete null hypothesis, and since the FDR of SGoF is not keeping any a priori level, it seems interesting to provide with an estimate of the FDR committed after the adjustment. In addition, the difference between the observed and expected proportions of *p*-values below γ may be more informative at threshold values other than γ = 0.05 [7], [10]. Therefore, in this work we are performing a double task. First, we present a new metatest method, called SGoF+, which is a modification of the SGoF previous algorithm taking advantage of the information on the p-values distribution in order to fix the rejection region. SGoF+ uses, as a discriminant rule, the maximum distance between the uniform distribution of *p*-values and the observed one, to set the null hypothesis for the binomial test (i.e. to choose γ). This maximizer corresponds to the Youden index for the classification problem, which means that it gives the maximum separation between the true positive rate and the false positive rate (an optimum in the ROC analysis). This new approach has a significant improvement on power over the previous method. Second, after applying SGoF+ or other adjustment method, we compute the associated *q*-values. The key point is that using a given *q*-value cutoff to decide what null hypotheses should be rejected is arbitrary. In fact, we can see SGoF and SGoF+ as metatest methods that lead to a ‘reasonable’ pFDR (*q*-value cutoff), because there is no a priori indication of the proportion of false discoveries one should respect. In what follows we will perform simulation of two-tailed one-sample t-tests to compare statistical power and pFDR after applying Sequential Bonferroni (SB), Benjamini-Hochberg (BH), SGoF and SGoF+ methods. The formalization of the new SGoF+ method is detailed in the Materials and Methods section. We have also compared up to four different pFDR estimation methods to see how well they perform under the different adjustments. We illustrate the new method with a reanalysis of a data set from a protein expression experiment in eggs of the marine mussel *Mytilus edulis* [8]. The new proposed SGoF+ method and the estimation of *q*-values have been incorporated in the last update of the program SGoF+ [9].

## Results

### Statistical power

**Figure 1:**
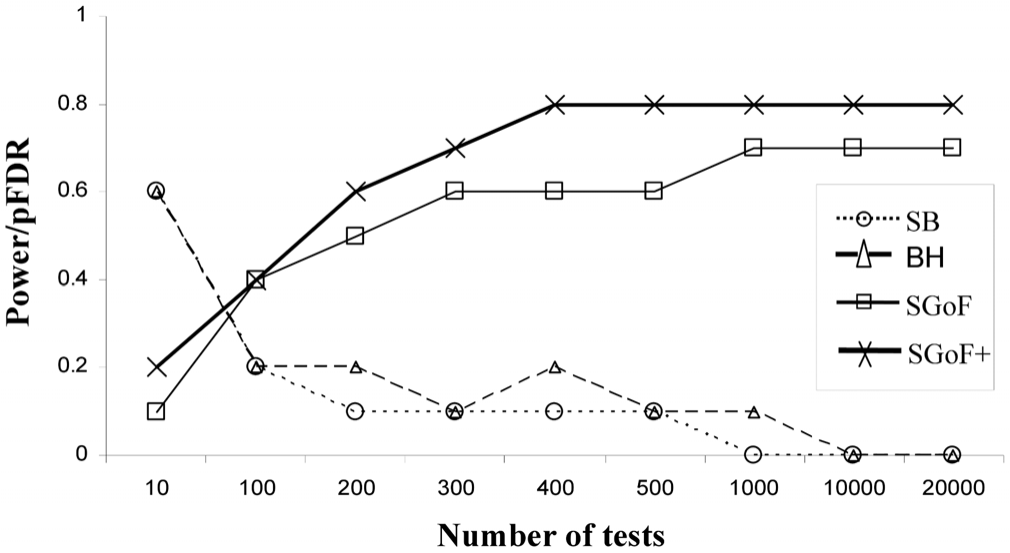
Power/pFDR ratio with different number of tests. The family of tests was 1,000 one-sample *t* tests with 20% of them coming from a N(0.36, 1) and sample size 20. Values are averages through 1,000 replicates.

To check the relationship between power increase through the number of tests and the pFDR committed we measured the ratio Power/pFDR. In the Figure 1 it can be appreciated that with a sample size of 20 BH (performed at FDR 0.05) and SB methods (performed at strong FWER control of 0.05) has a ratio larger than that of metatests SGoF (γ = 0.05) and SGoF+ (both performed at weak control of FWER of 0.05) only under the lowest number of 10 tests. The ratio Power/pFDR shows a decreasing shape through the number of tests for SB and BH methods, while the opposite is true for SGoF and SGoF+ with the latter showing the best ratio. The same pattern is obtained when the sample size is as low as 5 though in this case the effects are not so easily detected by SGoF and SGoF+ accordingly to the loosing of sample information (not shown). This Figure 1 reveals the practical superiority of SGoF+ with respect to the original SGoF method, at least when the goal is to increase the statistical power. Indeed, SGoF+ can be regarded as an automatic algorithm that finds inside the SGoF's family the γ leading to the maximum power and the minimum false non-discovery rate [10]. Of course, when doing that, an extra rate of false discoveries is committed, but the behavior of the quotient Power/pFDR indicates that this is compensated through the power increase.

### True positive versus false positive rate through different percentage of effects

**Figure 2:**
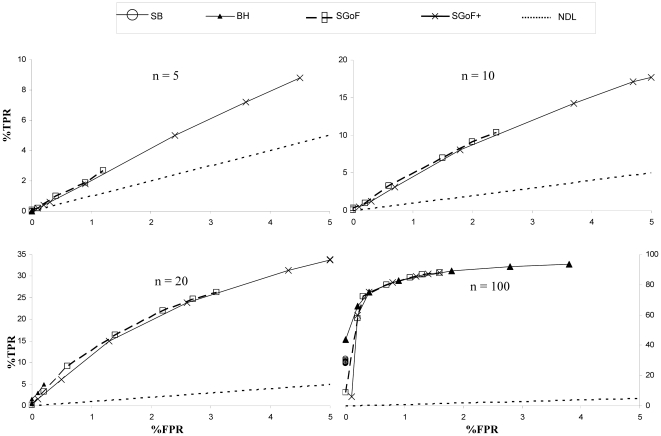
True versus false positives through different percentage of effects. The family of tests was 1,000 one-sample *t* tests. TPR: True Positive Rate. FPR: False Positive rate. Marked points in the lines represent 1, 5, 10, 20, 40, 60 and 80% of effects coming from a N(0.36, 1). *n*: Sample size. Values are averages through 1,000 replicates.

We have plotted the true positive rate (i.e. sensitivity, y-axis) against the false positive rate (1-specificity, x-axis) through different percentage of effects from 1 to 80% for the distinct multiple testing methods when the number of tests is 1,000 (Figure 2). Note that this Figure 2 displays a ROC curve in which different points are obtained according to the threshold value provided by each of the multiple testing adjustments, the points more to the left corresponding to the smaller proportions of effects. In particular, the x-axis varies from 0% to 5% since all the methods gave thresholds below 0.05. Obviously, the best possible method would yield points in the upper left corner or coordinate (0, 100). Values below the diagonal or no-discrimination line (NDL) would be considered as poor performing methods. Different panels for separate sample size cases are shown for different proportions (*% Effect* from 1 to 80%) of the alternative hypothesis contributing to the family of 1,000 comparisons (see Methods). The pattern for families of 10,000 comparisons was similar (not shown). Note that the y-axis scale varies among the distinct panels. It can be appreciated that SB method only has a true positive rate in the case of highest sample size (*n* = 100). The BH method needs sample size of 20 or higher to display a true positive rate, and it is the best method with *n* = 100 within the region of 1 to 10% of effects. With more than 20% of effects the true positive rate is almost constant while the false positive rate is increasing. With low sample sizes of *n* = 5 and *n* = 10 the only methods performing well are the metatest ones (SGoF and SGoF+), both of them being always far above the NDL except for sample size 5 and just 1-5% of effects. Thus, if we look to the y-axis (true positive rate), the BH and SB methods had no true positives (no power) under these sample sizes. Furthermore, we see that SGoF+ appears with larger *y* and *x* coordinates than SGoF, i.e. SGoF+ has true positive rate larger than that of SGoF although at the cost of increasing its false positive rate.

Interestingly, when sample size is the largest (*n* = 100) the metatest methods perform the best in the case of high percentage of effects (40% or higher). On the contrary BH performs the best with percentage of effects as low as 1% (first triangle from left to right with *n* = 100). This good behavior of BH with large sample sizes is not surprising from previous simulation results [6]; note also that a large sample size results in a strong relative effect, a situation in which FDR-controlling strategies are expected to perform well [10].

### Positive false discovery rate

**Figure 3:**
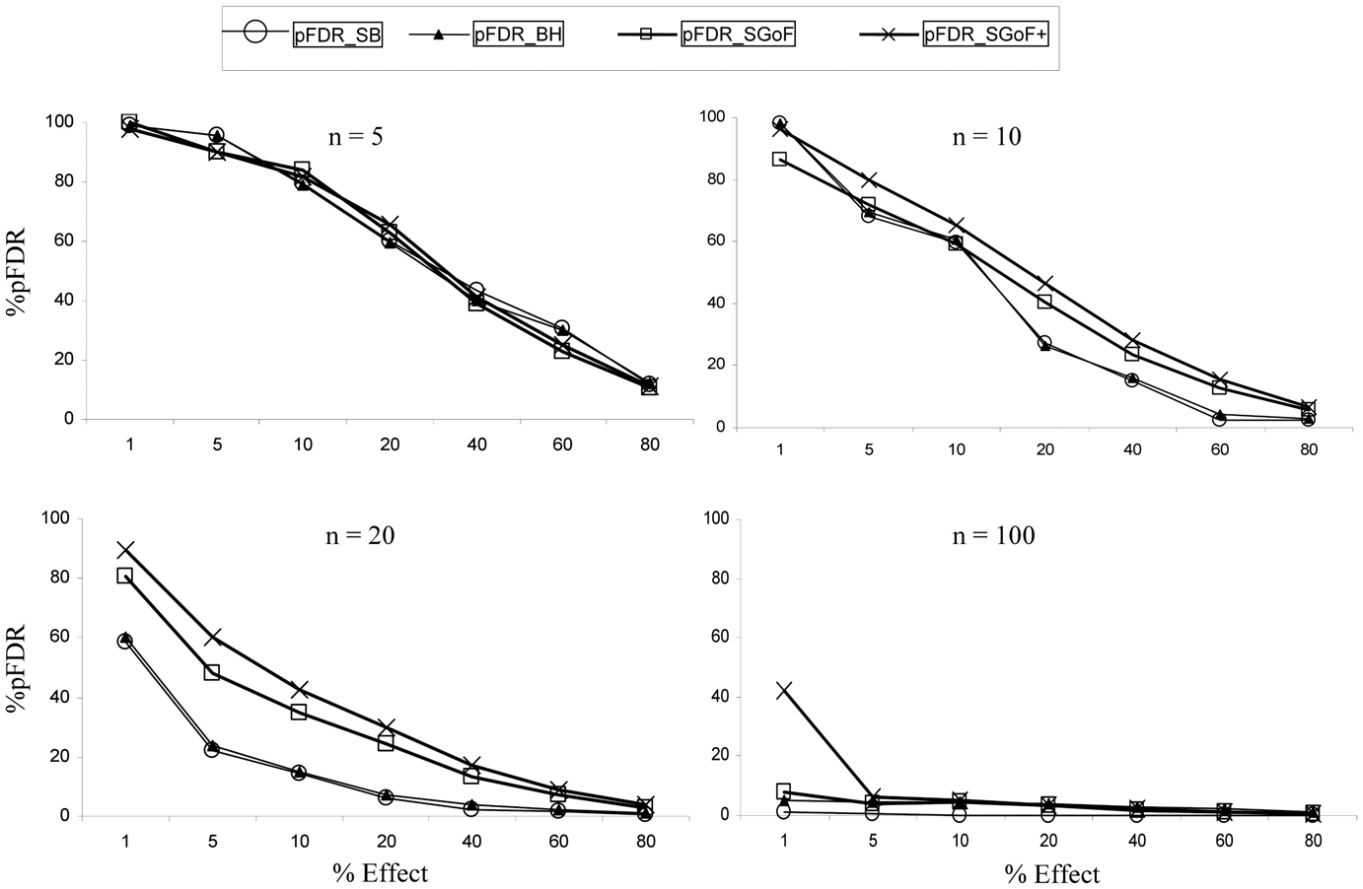
Observed positive False Discovery Rate (pFDR). The family of tests was 1,000 one-sample *t* tests with 1, 5, 10, 20, 40, 60 and 80% of them coming from a N(0.36, 1). *n*: Sample size. Values are averages through 1,000 replicates.

Because we are performing simulations we can exactly measure the false discovery rates committed by the different methods. The positive false discovery rate, pFDR, was measured and averaged through replicates. The difference of measuring pFDR instead of FDR is that when measuring FDR the average is taken through all runs including those without discoveries which will have a FDR of 0. However, in the case of pFDR only runs with discoveries are averaged. Upon inspecting Figure 3 we can firstly appreciate that the pFDR is decreasing with the increasing percentage of effects. This is expected since given that a discovery is reported (the necessary condition for measuring pFDR) the probability of it being a true one is higher with a larger number of true effects. Secondly, we can see that, independently of the method and of the percentage of effects, the pFDR is always higher with the lower sample size. This occurs because with larger sample size the relative effect is stronger in the sense that, for a given effect size, that effect will be more easily detected with a larger *n*. For the lowest sample size (*n* = 5) all correction methods perform similarly. In the other cases, the metatest methods have in general a larger pFDR than SB and BH especially with *n* = 20. With the largest sample size (*n* = 100) all methods perform similar again but the SGoF+ in the case of 1% of effect commits the highest pFDR.

### Estimation of positive false discovery rate

**Figure 4:**
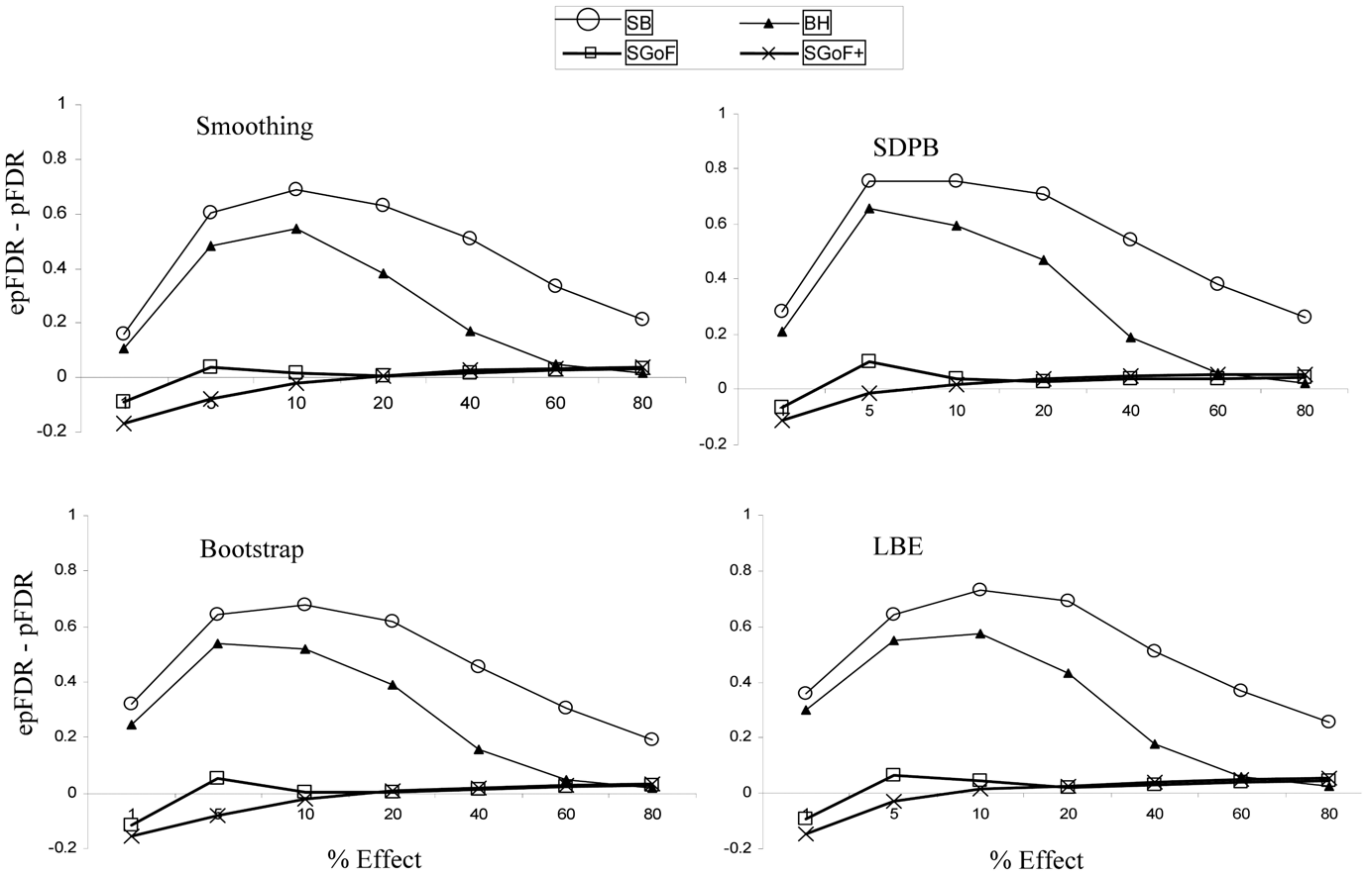
Estimated positive false discovery rate epFDR minus the observed pFDR. The family of tests was 1,000 one-sample *t* tests with 1, 5, 10, 20, 40, 60 and 80% of them coming from a N(0.36, 1). Sample size was 20. The panels: Smoothing, SDPB, Bootstrap and LBE, refer to the corresponding method to estimate the pFDR (see Methods section). Values are averages through 1,000 replicates.

To assess the performance of the several pFDR estimation methods (see Methods section) we plotted the difference between the estimated (epFDR) and the observed pFDR. Thus, the positive differences indicate conservative estimates. The results are given in Figure 4 for the case with sample size 20. We can appreciate that the four estimation methods perform similarly. Indeed, for the metatest methods (SGoF and SGoF+) the pFDR is accurately estimated always when the percentage of effects is above 1%. In the case of a number of effects as low as 1% epFDR slightly underestimate the observed pFDR corresponding to the metatests. For the SB and BH methods the pFDR estimates are quite conservative. The explanation to this behavior is that we are using the robust pFDR estimate [11] which grossly overestimates the pFDR when the probability of having a true effect is low.

### Example of application

We performed an example of application of SGoF+ jointly with the estimate of the *q*-values using a list of *p*-values coming from protein expression experiments in eggs of the marine mussel *Mytilus edulis* [8]. In that study, *M*. *edulis* female protein expression profiles of two lines differing in sex ratio of their progeny were compared. In that exploratory study authors had no power to detect any significant effect after correction with BH at the 20%. However, when proteins spots were able to be identified by mass spectrometry, authors briefly speculate about the possible biological role of some differences and so they decided to accept the necessary FDR to get the whole set of the a priori significant p-values (26 out of 261 tests at the 5% level). Therefore they assumed an FDR of 50% to get all 26 candidate spots [8]. Because that study had low sample size (two biological replicates in each group) and power it is an interesting one to check how many significant tests are detected and the FDR that will be committed using SGoF+.

We have used the SGoF+ software [9] in its last version, to apply metatest corrections jointly with the *q*-value estimate over the same 261 *p*-values from the Diz et al. [8] study. We have estimated the proportion of true nulls π_0_ using the four previously checked methods. We can appreciate (Table 1) that the four methods give different estimates with the SDPB giving the most conservative estimate (92%) while the Smoothing the most liberal (61%). When grouping these values in intervals of 0.05 length the mode is 0.82. Therefore, we use this modal value of 82% as our estimate of π_0_ to study which proteins should be considered as differentially expressed after correction with SGoF+ jointly with the consideration of the associated *q*-values (Table 2). Using a 5% significance level SGoF (γ  = 0.05) detects 6 spots with associated *q*-value of 0.22 corresponding to the highest significant *p*-value (0.007) The discriminant rule of SGoF+ automatically gives γ = 0.244703 and SGoF+ detects 17 significant protein spots with *q*-value of 0.32 for the highest significant *p*-value (0.026). Concerning to our π_0_ modal estimate if we decide to reject all 26 spots with *p*-values below 0.05 we should assume an FDR of 40% instead of the 50% assumed in Diz et al. [8] study. We also performed the analysis at 0.1% significance level to get just 1 significant spot after SGoF+ with a *q*-value of 0.2 (Table 2). In this case the number of effects declared by SGoF+ is limited to 1 because there is only one *p*-value below 0.001 among the 261 original ones.

**Table 1:** Proportion, π_0_, of truly null features estimated by different methods for the dataset of Diz et al (2009).

Method	π_0_
Bootstrap	0. 80
LBE	0. 84
SDPB	0. 92
Smoothing	0. 61
Mode	0.82

The mode of the methods is computed using intervals of length 0.05 for grouping the data.

**Table 2:** Number of significant tests after multiple test adjustment at the 5% and 0.1% levels and the q-value associated to them for the dataset of Diz et al (2009).

Method	# of tests 5%	*q*-value	# of tests 0.1%	*q*-value
SB	0	---	0	---
BH	0	---	0	---
SGoF	6	0.22	0	---
SGoF+	17	0.32	1	0.20

## Discussion

There is a problem with multiple test adjustment methods trying to control type I error rates because of the increase of the type II error i.e. the loss of power when the number of tests is high. Furthermore, it is known that the methods controlling FDR are not controlling pFDR in some situations [2], [5]. It seems clear that pFDR is what the researcher desires to control at least if interest is restricted to experiments where some discovery has been made [12]. So, when applying an FDR-based adjustment as BH we are loosing power while still not necessarily controlling false discoveries as desired.

In fact, we have shown (Figure 3) that SB, BH and the metatest methods commit very similar pFDR under realistic sample size (*n* = 5). This is interesting since the power of SGoF+ method is the highest so, its power/pFDR rate will be better.

Therefore, it is reasonable to take advantage of a priori information on the *p*-value distribution and perform the estimation of the *q*-values linked to the *p*-values, i.e. the pFDR we expect to commit over all the rejection regions below. Indeed, if sample size is low and the effects are weak, the robust pFDR estimation method [11] should be preferred. In any case, the use of the *q*-values does not provide an automatic procedure which maximizes the power of detecting true effects while informing about the probability of committing a false discovery. We have shown that metatest methods as SGoF and SGoF+ represent a good compromise between power and pFDR (Figure 1) when the number of tests is larger than 100. Importantly, results obtained for SGoF+ indicate that this modification reports an extra power, which compensates for the resulting increase in the proportion of false discoveries.

Thus, we suggest combining SGoF+ with the information provided by the *q*-values as a reasonable tool to perform the multiple test adjustment.

The proposed strategy was illustrated by analyzing real data from a protein expression experiment. In the original study [8] the authors faced the question of what FDR should be assumed in order to get some positives to continue with their exploratory analysis. Because a FDR of 20% does not produced any positive they decided to assume the cost of a 50% FDR to get the whole set of 26 positives obtained previously to the multiple testing correction. From an exploratory point of view such a low number of candidates represents a good cost-benefit compromise for further confirmatory studies (Diz, personal communication). Here we applied SGoF+ to conclude that there is statistical motivation to assume 17 positives as significant. Hence the FDR to assume in doing so is provided by the corresponding q-value estimate of 0.32 so we expect 11–12 out of those 17 to be the true positives. One interesting point about SGoF+ is the kind of statistical significance the method searches for. In general, the number of significant cases provided by the SGoF strategy can be regarded as a lower bound for the number of effects with p-value smaller than γ [10]. For the protein expression experiment, the discriminant rule gave γ = 0.24; then, SGoF+ is telling us that there are at least 17 tests among the 94 tests with p-value below 0.24 which correspond to true effects. This kind of evidence is useful in settings where FDR-controlling strategies suffer from a remarkable lack of power.

An important issue related to multiple testing in high-throughput experiments is the intrinsic inter-dependency in gene effects [13]. Usually weak-dependence, which corresponds to local effects between a small number of genes, is considered [14]. It has been shown that under weak-dependence, both the FDR-based [4], [14] and the SGoF [6] methods are still robust. Indeed we have checked that in the worst case of no effects (complete null hypothesis) and with pair wise correlations of 0.1 the FWER is being controlled by SGoF+ still when blocks of correlated genes are of size 100. When correlations are as large as 0.5 then blocks of size 50 provoke the loosing of the FWER control of SGoF+ but the number of false positives is still very low [15]. The behavior of SGoF+ under dependence is still better when some percentage of true effects exist (data not shown). Since short blocks of correlated genes are expected in genome and proteome wide studies [4], [13] the above methods should even be useful. However, in some cases high correlations could be found in the data and this could have great impact in the *p*-value distribution and consequently in the correction methods [16]–[17]. In the case of the metatest correction methods, SGoF and SGoF+, such high correlations could provoke the weak FWER control to be lost. The BH however still remains conservative as expected when the dependence relationships are positive [18]. Therefore, if strong dependence is suspected the combination of metatest methods with the most conservative BH method should be preferred. Alternatively the empirical null distribution can be computed to get the adequate critical level for the multiple testing [16]. An automatic correction of metatest methods for data with strong dependence is work in progress.

Finally, it is worth mentioning that the above adjustment methods and the *q*-value estimation have been implemented in the latest version of the SGoF+ software. The *q*-value estimation is performed via the different methods used in this paper, for the robust and standard pFDR estimation (*C* parameter in formula (2), see Methods). The program provides to the user with two files. The first one includes a list of selected tests after performing the adjustment by each method at a desired significance level. The second one (in excel and html formats) provides the user with the full list of a priori significant tests and the adjusted *p*-values for each correction method, jointly with the estimated *q*-value for each test.

As a conclusion, it seems that SGoF+ shows an improvement in the statistical power to detect true effects with respect to other adjustment methods including SGoF. Combining SGoF+ with the *q*-value associated to each test can be an interesting strategy when performing multiple test adjustments. The latest version of the SGoF+ software is freely available at http://webs.uvigo.es/acraaj/SGoF.htm.

## Materials and Methods

### Formalization of the SGoF+ test

Consider testing at significance level *γ* a set of *S* null hypotheses *H_1_, H_2_, …, H_S_*. Let *p*_1_≤*p*_2_ ≤…≤ *p*_S_ be the sorted *p*-values, and denote by *H*_i_ the null hypothesis corresponding to *p*_i_ . Individually, each null hypothesis is rejected when the *p*-value is smaller than the given *γ*. However, all these rejections can not be identified as true effects since the individual tests do not correct for the multiplicity of tests. Let *K_γ_* be the proportion of rejections with such a procedure. Provided that the *S* nulls are true, the expected proportion of rejections (i.e. false positives) is *E*(*K_γ_*) = *γ*. Consider now from a set of possible *γ* values the one which maximizes the difference between the observed and the expected proportions of rejections, i.e. *γ*_ 0_ =  arg max *_γ_* { *K_γ_* – *E*(*K_γ_*)} = arg max*_γ_* { *K_γ_* – *γ* }.

We perform a goodness-of-fit test via an exact binomial test or (for S≥10) a chi-squared test with one degree of freedom onto the null hypothesis H_0_: *E*(*K_γ_*_0_) = *γ*_ 0_ at a desired level α. The procedure now is identical as in the previous SGoF version [6]. Let *b*_α_(*γ_0_*) be the critical value of *S×K*_γ0_ for such a goodness-of-fit test (i.e. *b*_α_(*γ_0_*) is the 100(1-α)% percentile of the *Binomial(S,γ_0_)* distribution); that is, the test gives rejection at level α when *S×K_γ0_*≥*b*_α_(*γ_0_*). Here, “rejection” means that at least one of the null hypotheses is false. In the case of rejection, the test concludes that the *N_α_(γ_0_  =  min(S×K_γ0_ - b*_α_(*γ_0_*) +*1*, *S× K_α_*) hypotheses with the smallest *p*-values (these are, *H_1_ , H_2_ ,…, H_Nα(γ0)_*) are false. This is a subset of the initial set of *S× K_α_* hypotheses one would reject when performing the *S* tests individually at level α. We bound the ‘excess of significants’ *S×K_γ0_ - b*_α_(*γ_0_*) *+1* in the metatest by *S× K_α_* in the definition of *N_α_(γ_0_)* to exclude the *p*-values above α as potential discoveries. This caution was not needed in the original conception of SGoF which just sets γ_0_ = α (so *S×K_γ0_ - b*_α_(*γ_0_*) *+*1<*S× K_α_* in this case).

It has been shown [6] that the SGoF method controls for FWER in the weak sense at the α level; however, the automatic selection of γ_0_ introduced by SGoF+ gives a FWER above the nominal. This problem is avoided by adding a preliminary step to compare the *K*_γ0_ - *E*(*K*_γ0_) with the critical value of the one-sided Kolmogorov-Smirnov test at level α, *ks_α_* , so no effect is declared when *K*_γ0_ - *E*(*K*_γ0_)<*ks_α_*. By definition of γ_0_, this correction (which has been incorporated in the implementation of SGoF+) guarantees a family wise error rate of 100α% (Figure S1). Note that the one-sided Kolmogorov-Smirnov test is adequate because it computes the supremum of the set of distances between the theoretical (the uniform) and the empirical distribution function of the *p*-values.

### True positive versus false positive rate through different percentage of effects

True positive rate (TPR) is expressed as the power or sensitivity, i.e. proportion of true effects which were correctly identified. False positive rate (FPR) is expressed as the fraction of false positives out of the negatives i.e. one minus specificity, where specificity is the proportion of nulls which were correctly identified. Plots of TPR versus FPR are computed for different number of tests (*S* = 1,000 and 10,000), sample sizes *n* = 5, 10, 20, 100 and percentage of effects. Specifically, the points in Figure 2 are computed as TPR versus FPR for 1, 5, 10, 20, 40, 60 and 80% of tests having effects (alternative hypothesis).

### pFDR estimation (epFDR)

The proportion π_0_(λ) of features that are null and the point λ from which the uniform distribution of *p*-values occurs are estimated via four different methods.

#### 1.- Smoothing

We fit a natural cubic spline to the data (λ_r_, π_0_(λ_r_)), where λ_r_, *r*  = 1,...,*R*, is a grid of λ-values and π_0_(λ_r_) follows formula (1) below, and we evaluate it at the point λ = 1 to get the π_0_ estimate [4]. The algorithm is that in Storey and Tibshirani [4] except that the number of degrees of freedom is not limited to be 3. By default we use the grid λ_r_ = 0, 0.05, 0.1,…, 0.95.

#### 2.- Bootstrap

We estimate the point λ by minimizing the mean-squared error of the estimated π_0_. This is attained via bootstrapping the *p*-values. This is exactly the same procedure as in Storey [14]. Once that λ is estimated, the proportion of true nulls π_0_ is computed as 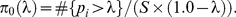
(1)


#### 3.- LBE

The location based estimator (LBE) of π_0_ proposed in Dalmasso et al [19]. A threshold *l* = 0.05^2^ for the variance upper bound of the estimator was assumed so that, *m* = 1 for 2≤*S*<2000, *m* = 2 for 2000≤*S*<7500 and *m* = 3 for *S*≥7500 where *m* are the natural numbers corresponding to *n* in Dalmasso et al [19].

#### 4.- SDPB

The method proposed in Meinshausen and Rice [20]. Estimating the proportion *L* of false null hypotheses can be achieved by bounding the maximal contribution of true nulls to the empirical distribution function of *p*-values. We use the standard deviation-proportional bounding function that has been shown to have optimal properties among a large class of possible bounding functions [20]. The proportion of true nulls is computed as π_0_ = 1-*L*.

Once the proportion of true null hypotheses π_0_ is estimated by any of the methods above, the estimated pFDR (epFDR) is computed as
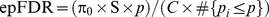
(2)where *p* is the p-value threshold of a given multitest correction and *C* = 1- (1 - *p*)^S^. This is the robust pFDR estimation given in Storey [11]. The standard, i.e. non-robust, pFDR estimation is attained just by setting *C* = 1 in (2).

To compare the performance of the above pFDR estimations we measure the difference between the estimated epFDR and the observed pFDR. In the real data example section we have assigned the different π_0_ estimates to intervals of length 0.05 in order to compute π_0_ as the mode of the different estimates.

### Simulations

To compare the efficiency of the proposed new SGoF+ metatest jointly with the precision of the pFDR estimation methods, we performed one sample two-tailed *t*-tests. As in previous work [6], we implemented a modification of the procedure outlined in Brown and Rusell [21] to perform the series of *t* tests.

For a given sample size *n*, we got a sample of *n* standard normal deviates N(0,1) if there is no effect or N(*x*,1) if there is an effect of size *x*. After that, we performed the *t*-test for the null hypothesis that the sample belongs to a population with mean 0 and variance 1 which is true if there is no effect but false otherwise. The *t*-test values were transformed to *p*-values via the incomplete beta function [22]. In this work we focused in the case with weak effects. That is, while the mean of the normal deviates generated for the null hypothesis was 0, we chose the mean for the alternative hypothesis so that the probability of a *p*-value less than 5% should be 0.10 under asymptotic conditions. This means an effect of *x* = 0.36 i.e. sampling from N(0.36,1).

We assayed different percentages (% effect = 1, 5, 10, 20, 40, 60 and 80%) for the alternative model being true with respect to the total number *S* of tests. We generated the normal deviates under a given, null or alternative, distribution, with sample size *n* = 5, 10, 20 or 100. Because we performed a two-tailed *t-*test with *n* -1 degrees of freedom, at the 5% significance level, there was a power of 0.10, 0.18, 0.33 and 0.95 respectively, for the sample sizes indicated above, when we tested versus the alternative with mean 0.36. These were, at each test, the probabilities for rejecting the null being false i.e. detecting true effects. Each test case was replicated 1000 times to obtain empirical standard deviations in the estimates.

## Supporting Information

Figure S1 **Family Wise Error Rate (FWER) with different number of tests.**
(TIF)
