# Supplementary figures and images for: Assessing Significance in High-Throughput Experiments by Sequential Goodness of Fit and q-Value Estimation
Source: PLoS One. 2011 Sep 9;6(9):e24700. doi: 10.1371/journal.pone.0024700 (PMC3170371; doi:10.1371/journal.pone.0024700)

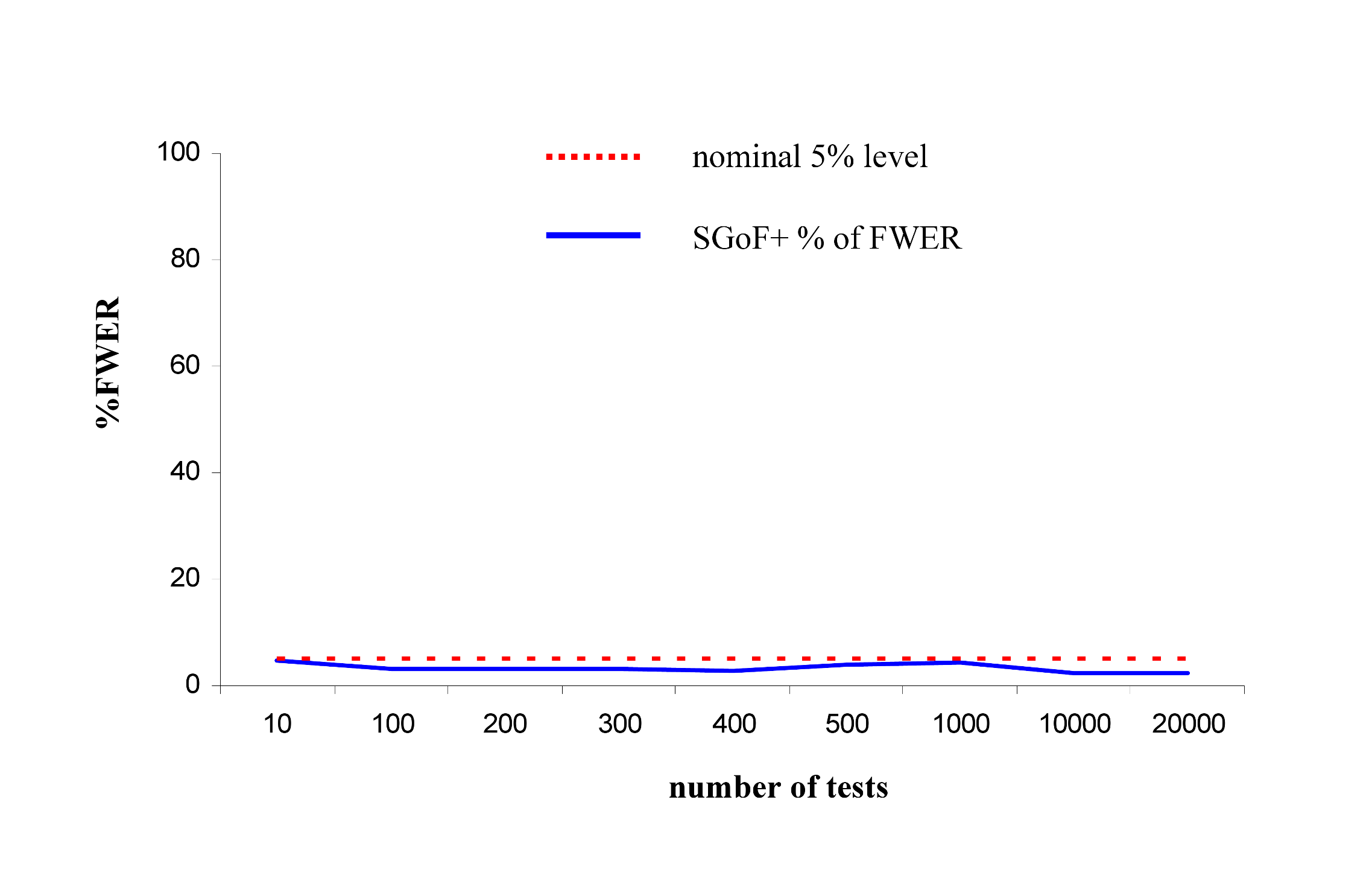

Supplement: Figure S1 — Family Wise Error Rate (FWER) with different number of tests. (TIF) [file pone.0024700.s001.tif]
